# Favourable neurological outcome following paediatric out-of-hospital cardiac arrest: authors’ reply

**DOI:** 10.1186/s13049-024-01176-3

**Published:** 2024-01-26

**Authors:** Alexander Fuchs, Roland Albrecht, Robert Greif, Martin Mueller, Urs Pietsch

**Affiliations:** 1 Department of Anaesthesiology and Pain Medicine, Inselspital, Bern University Hospital, University of Bern, Freiburgstrasse, 3010 Bern, Switzerland; 2 Unit for Research in Anaesthesia, Department of Paediatric Anaesthesia, IRCCS, Istituto Giannina Gaslini, Genova, Italy; 3 Swiss Air-Ambulance (Rega), Zurich, Switzerland; 4 Department of perioperative Intensive Care Medicine, Cantonal Hospital St. Gallen, St. Gallen, Switzerland; 5 Faculty of Medicine, University of Bern, Bern, Switzerland; 6 School of Medicine, Sigmund Freud University Vienna, Vienna, Austria; 7 European Resuscitation Council (ERC) Research NET, Niel, Belgium; 8 Department of Emergency Medicine, Bern University Hospital, Inselspital, University of Bern, Bern, Switzerland

We thank Benjamin Stretch [1] for commenting on our article[2] in which we investigated factors associated with favourable neurological outcome in paediatric patients sustaining out-of-hospital cardiac arrest (CA).

Considering all helicopter emergency service system (HEMS) missions in Switzerland, paediatric out-of-hospital CA is a very rare condition [3]. We agree that traumatic CA substantially differs from non-traumatic CA. Given the large proportion of traumatic CA in our analysis, we felt the importance of including both aetiology as it reflects real-life data of a high-performing national HEMS. Indeed, survival with favourable neurological outcome was significantly higher in patients with non-traumatic CA (*n* = 53/199, 26.3%) compared to traumatic CA (*n* = 3/97, 3.1%) [2].

We thank the author for mentioning the 2021 European Resuscitation Council (ERC) guidelines for CA in special circumstances [4]. However, the mentioned guidelines cover adult patients. In Switzerland, patients up to 16 years sustaining out-of-hospital CA are treated in specialised paediatric hospitals [5], which was the rationale for our age limitations. Unfortunately, the applicable guidelines on Paediatric Life Support [6] only recommend adjusting the algorithm in special settings (e.g. trauma). The very brief chapter on Traumatic CA in the ERC Paediatric Life Support guidelines [6] recommends starting standard CPR while searching for and treating reversible causes. These guidelines [6] suggest that the interventions for reversible causes might precede adrenaline administration. We agree that performing chest compressions in patients with severe exsanguination, tension pneumothorax, or cardiac tamponade might be useless. However, no specific algorithm is provided in the guidelines [6] to aid decision-making for clinicians in these stressful situations. Swiss HEMS physicians are trained in paediatric advanced trauma life support, including traumatic CA.

The analysis of our data showed that favourable neurological outcome was higher for patients receiving immediate bystander CPR before HEMS treatment. We don’t consider ground-based ambulances competing HEMS. The latter poses additional advantages to the whole performance of the emergency medicine system. Indeed, HEMS offers the possibility to bring highly experienced personnel fast on-scene, which might be an advantage as potentially life-saving critical interventions can be performed early [3]. Other important factors in the chain of survival include the critical phase of post-resuscitation care. HEMS physicians can initiate state-of-the-art post-resuscitation care already on-scene and during the flight into specialised paediatric hospitals with adjunct operating rooms and intensive care units. These specialised paediatric hospitals [5] can be reached by helicopter fast.

However, such retrospective data analysis should be carefully interpreted as the association does not imply causality. Several of these limitations are discussed in our article [2].

Finally, we highly appreciate the awareness campaign *‘Aaron’s Heart’* of the Resuscitation Council United Kingdom to improve outcomes for paediatric CA patients.

## Data Availability

Not applicable.

## Abbreviations

CA: Cardiac Arrest
CPC: Cerebral Performance Category
CPR: Cardiopulmonary resuscitation
HEMS: Helicopter Emergency Medical Service
ERC: European Resuscitation Council
